# Broadband Electrical Spectroscopy to Distinguish Single-Cell Ca^2+^ Changes Due to Ionomycin Treatment in a Skeletal Muscle Cell Line

**DOI:** 10.3390/s23094358

**Published:** 2023-04-28

**Authors:** Caroline A. Ferguson, Carmen Santangelo, Lorenzo Marramiero, Marco Farina, Tiziana Pietrangelo, Xuanhong Cheng

**Affiliations:** 1 Department of Bioengineering, P.C. Rossin College of Engineering and Applied Sciences, Lehigh University, Bethlehem, PA 18015, USA; 2 Department of Neuroscience, Imaging and Clinical Sciences, University “G. d’Annunzio” Chieti-Pescara, 66100 Chieti, Italy; 3 Department of Engineering of Information, University Politecnica delle Marche, 60131 Ancona, Italy; 4 Department of Materials Science and Engineering, P.C. Rossin College of Engineering and Applied Sciences, Lehigh University, Bethlehem, PA 18015, USA

**Keywords:** broadband impedance spectroscopy, circuit modeling, single-cell characterization, intracellular calcium

## Abstract

Many skeletal muscle diseases such as muscular dystrophy, myalgic encephalomyelitis/chronic fatigue syndrome (ME/CFS), and sarcopenia share the dysregulation of calcium (Ca^2+^) as a key mechanism of disease at a cellular level. Cytosolic concentrations of Ca^2+^ can signal dysregulation in organelles including the mitochondria, nucleus, and sarcoplasmic reticulum in skeletal muscle. In this work, a treatment is applied to mimic the Ca^2+^ increase associated with these atrophy-related disease states, and broadband impedance measurements are taken for single cells with and without this treatment using a microfluidic device. The resulting impedance measurements are fitted using a single-shell circuit simulation to show calculated electrical dielectric property contributions based on these Ca^2+^ changes. From this, similar distributions were seen in the Ca^2+^ from fluorescence measurements and the distribution of the S-parameter at a single frequency, identifying Ca^2+^ as the main contributor to the electrical differences being identified. Extracted dielectric parameters also showed different distribution patterns between the untreated and ionomycin-treated groups; however, the overall electrical parameters suggest the impact of Ca^2+^-induced changes at a wider range of frequencies.

## 1. Introduction

The concentration of calcium (Ca^2+^) ions in the context of muscle cell operation has several complex and dynamic functions. The Ca^2+^ within the cytoplasm skeletal muscle cells has a variety of deeply important functions, including regulating the mitochondrial function and controlling signaling pathways between cells. In skeletal muscle systems, Ca^2+^ has roles in the excitation–contraction (EC) coupling, mitochondrial function, and as a signal regulator for protein and degradation [1,2,3]. These functions are carefully controlled by the rhythmic movement of Ca^2+^ from storage in the sarcoplasmic reticulum (SR) to the cytosol, fostered by a variety of well-characterized proteins in skeletal muscle cells [4,5]. As mentioned, Ca^2+^ plays a continuous role in muscle contractibility and the development of mitochondrial abnormalities leading to diseases mostly characterized by symptoms involving loss of muscle control and fatigue [6]. Natural muscle structure changes with age, named sarcopenia, or as the result of a genetic disease such as Duchenne Muscular Dystrophy (DMD), or atrophy resulting from cancer cachexia or acquired immunodeficiency syndrome (AIDS) [4,5,7,8]. In these diseases, the increased cytoplasmic Ca^2+^ results in the eventual degradation of muscle filaments and proteolytic pathways that maintain muscle integrity and mitochondrial polarization that helps with energy management [9]. Therefore, the ability to distinguish Ca^2+^ changes intracellularly have an implication for potential diagnostic and therapeutic abilities.

Ions such as Ca^2+^ and other charged particles can impact the passage of intracellular charges and external electrical stimuli. Electrical diagnosis poses a promising method of characterizing single-cell cytoplasmic changes associated with diseases of muscular integrity and could develop a unique quantitative diagnostic and evaluative tool. Shifting to electrical measurement also offers the opportunity to adopt a rapid and label-free approach to future diagnoses. Developing a modeled understanding of how certain specific changes in disease progression in skeletal muscle cells impact cell impedance lays the groundwork for generating a disease-specific electrical profile that could be both tunable and predictive. Broadband spectroscopy measures cell impedance over a wide range of frequencies that have been prominent in distinguishing single-cell characteristics, especially in the last decade [10,11,12,13]. By studying a broad bandwidth of frequencies using spectroscopy, the impedance measurement can enable the characterization of different cellular compartments based on the scattering of the signal and dielectric properties of the compartment. Biological tissue and cells have dielectric properties of conductivity (*σ*) and permittivity (*ε*), whose main intracellular scattering influences are widely expanded upon in review articles [14,15,16]. Various studies have identified frequencies of interest for disease identification [17]. At frequencies in the megahertz range, the dominant scattering method is β-scattering, making the primary signal changes attributable to cytoplasmic changes at a single-cell level [16]. By combining this understanding of broadband measurements with electrical circuit modeling, we study the physiological and dielectric properties of two cell populations.

## 2. Materials and Methods

### 2.1. Cell Culture

**Figure 1:**
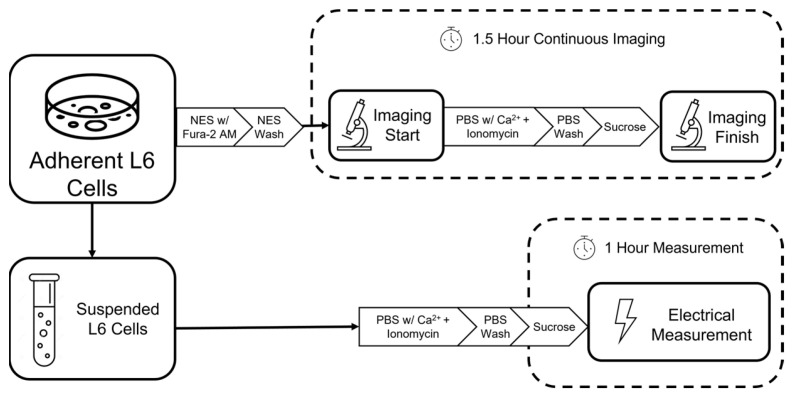
Experimental layout describing the biological characterization and parallel electrical measurements system.

L6 rat myoblast cells (ATCC, CRL-1458) were cultured according to American Type Culture Collection (ATCC) guidelines [18]. The cells were grown in Dulbecco’s Modified Eagle Medium (DMEM) base (Corning 10014CV, Corning, NY, USA) supplemented with 10% FBS (Gibco 10082147, Billings, MT, USA) and 5% Penicillin/Streptomycin (Life Technologies 15140122, Carlsbad, CA, USA) and incubated at 37 °C with 5% CO_2_. The media was refreshed regularly and cells were split when confluence on the flask surface was achieved. In Figure 1, the layout of how these adherent cells are treated and used for biological and electrical characterization is outlined.

### 2.2. Ionomycin Treatment

Cells were detached from the culture flasks and subjected to an Ionomycin treatment protocol previously established [19,20]. For electrical measurements, spun-down and detached cells were suspended in 1 µM Ionomycin (MilliporeSigma, Bedford, MA, USA, 56092-81-0) in a solution of phosphate-buffered saline (PBS) with Ca^2+^ (1.5 mM). The cells remained in this solution for 10 min at room temperature before rinsing with Ca^2+^-free PBS and preparation for electrical measurement in a solution of 8.5% sucrose and 0.3% dextrose. Similar steps were conducted on adherent cells after the introduction of the Ca^2+^ imaging agents, as shown in Figure 1.

### 2.3. Ca^2+^ Imaging

For measurements of the intracellular calcium concentration ([Ca^2+^]_i_), the cells were seeded in a 96-well plate in the culture media described previously at a density of 1000 cells per well for three days at 37 °C with 5% CO_2_. The cells were incubated with physiological solution containing (mM) NaCl 140, KCl 2.8, CaCl_2_ 2, MgCl_2_ 2, glucose 10, Hepes–NaOH 10, pH 7.4, named normal external solution (NES) containing 10 mg mL^−1^ bovine serum albumin and 5 µM Fura-2 AM (Molecular Probes, Eugene, OR, USA) for 45 min. Then, the cells were rinsed by incubation in NES with Ca^2+^ for 40 min at 37 °C to allow de-esterification of the dye inside the cells. Ca^2+^ imaging was performed using epifluorescence microscopy that assists in the acquisition of the Fura-2 emission fluorescence at both an excitation of 340 nm and then at an excitation of 380 nm. These excitations and measurements were accomplished using a Xenon lamp and monochromator wavelength switcher Polychrome II (Till Photonics, Gräfelfing, Germany) to isolate the wavelength of interest. The emission fluorescent images were collected using a 40× oil objective lens, acquired by an intensified CCD camera or a C6790 cooled CCD camera (Hamamatsu Photonics, Hamamatsu, Japan), digitized and stored in an interfaced computer. The software Aquacosmos (version 2.0) was used to analyze the resulting emission fluorescence ratio (f[340/380]) on selected representative cellular areas after the subtraction of background fluorescence. After an initial baseline was established, 1 µM Ionomycin in 1.5 mM Ca^2+^ was added for 10 min while measuring fluorescence. These measurements were accomplished in a temporal sequence generating spectral measurements showing the cell fluorescence ratio prior to ionomycin treatment, during treatment of 10 min, following a wash Ca^2+^-free PBS, and after incubation in an 8.5% sucrose and 0.3% dextrose solution. The mean initial values and post-treatment values were calculated using 25 time points in the initial plateau and the plateau following the wash, which were used to calculate the ratio. The acquisition time for each fluorescence emission was 0.5 s and the overall imaging lasted 1.5 h. Additional experiments were performed using sucrose solution with 10 mM EGTA, a calcium chelator, in order to prevent any possible external Ca^2+^ interference post-ionomycin treatment, showing similar results.

### 2.4. Electrical Measurement

**Figure 2:**
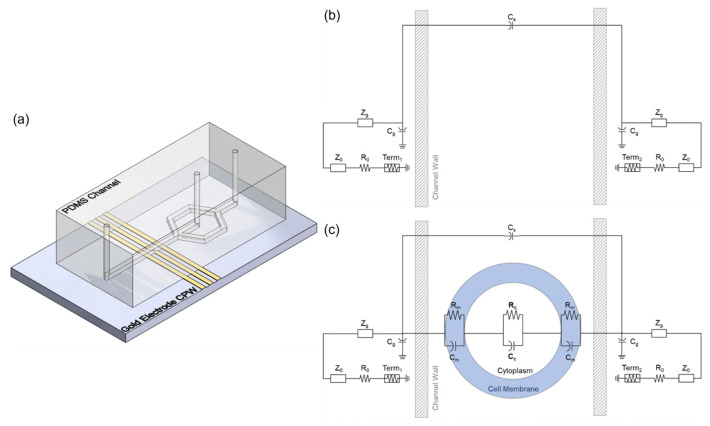
Schematics of (**a**) coplanar waveguide electrodes and PDMS channel, (**b**) background circuit model, and (**c**) single-shell circuit model.

Prior to cell preparation or treatment, a coplanar waveguide (CPW) electrode device was assembled by attaching a polydimethylsiloxane (PDMS) channel to the CPW surface aligned with a series gap. Further details on the CPW and channel design can be found in previous publications [21,22,23] and a simplified schematic can be seen in Figure 2. The assembled device was placed on a microscope stage and an isotonic solution of 8.5% sucrose and 0.3% dextrose in deionized water was passed through to test the assembly seal prior to the addition of samples. Cells treated with ionomycin and washed with Ca^2+^-free PBS were suspended in the same sucrose and dextrose solution at a concentration of 500,000 cells/mL and a sample was taken up in a syringe for injection into the channel assembly. Based on previous work, the cells remain their integrity for up to an hour, allowing continuous measurement.

Once all components were in place, cells were introduced to the channel at a sample flow rate of 0.1 µL/min along with a stream of sheath sucrose at a rate of 0.2 µL/min. An electrical dielectrophoretic (DEP) signal of −9 dBm at a frequency of 500 MHz was introduced between the series gap in the electrodes to trap the cell. Then, a broadband sweeping signal of −15 dBm from 9 kHz to 9 GHz was applied to measure the S-parameters with and without the cell present using a virtual network analyzer (Keysight E5080A, Santa Rosa, CA, USA). The S-parameters include the reflection coefficient (S11) and the transmission coefficient (S21). S11 is representative of the power reflected back to the power input port 1 by the system, while S21 is representative of the power that passes through the system to port 2 [24]. These measurements were repeated after the release of the previously trapped cell to determine the background measurement of the sucrose solution for these parameters. The differential S-parameters with and without the cell present, ΔS11 and ΔS21, represent the cell contribution to power reflection and transmission. The S-parameter spectra as a function of sweeping frequency were further fitted to a circuit model to extract impedance associated with the cell.

The raw signals of ΔS11 and ΔS21 taken from the Keysight network analyzer for each cell at all 201 frequencies appear as spectra. Using the impedance measurements for these spectra, curve fitting using simulated circuit modeling was used to obtain dielectric parameters.

### 2.5. Simulated Circuit Modeling

**Figure A1:**
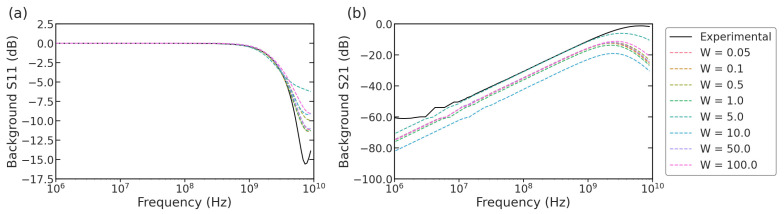
Background simulation fitting to experimental data for representative cell for (**a**) S11 and (**b**) S21 spectra.

**Figure A2:**
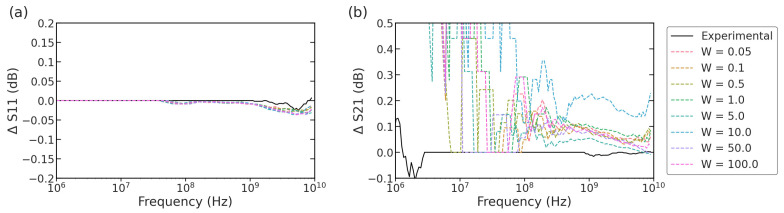
Delta simulation fitting to experimental data for representative cell for (**a**) ΔS11 and (**b**) ΔS21 spectra.

**Figure A3:**
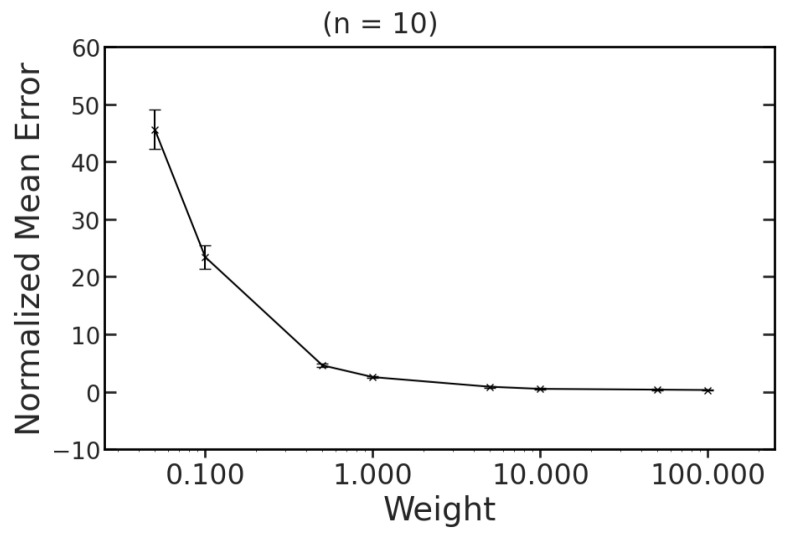
Normalized error for weights applied to Δ curve fitting optimization.

Simulation of a single-shell circuit model was performed to find the optimal fit of the cytoplasm resistance and capacitance for each electrically measured cell using Advanced Design System (ADS, version 2023.01) software by Keysight. The circuit designs for the simulations can be seen in Figure 2b,c. For each background file, variables were tuned to fit the properties of the electrode system and solution in a representative circuit to determine the best values to carry on matching the cell itself. Using the optimal background characteristics, a circuit modeling the cell layers of membrane and cytoplasm as resistor-capacitor pairs was used to find the best estimate of the cytoplasm properties. Based on previous measurements of L6 cells, the membrane resistance and capacitance were kept constant at 235 MOhm and 34.5 pF [25]. The fitting process was automated by treating the file number as a variable for each matching pair of background and cell measurement files in lists. The goal weights described in Table 1 were assigned by making a shared goal for each optimization and signal type and minimizing the error to fit all frequencies and a region of interest. The applied weights were determined by comparing the error generated by fitting a random smaller subset of spectra to best represent the background and spectra shape (Figure A1 and Figure A2). The chosen weight values to reduce the overall fitting error for the differential delta (Δ) spectra, which ended up being 5 times the background weights (Figure A3). The more specific and heavily weighted goals were applied to frequency ranges where the treated and untreated cells have the largest distinction in the S parameter spectra. These frequency ranges match theoretical understanding of the frequencies in which cytosol contribution dominates [26]. In addition, an overall weight increase was used to value the fit of ΔS11 to compensate for the smaller measured values.

For each pair, the background variables were tuned by minimizing the difference of the ranges described in Table 1 by applying the described goal and weight values. The fit variables were then applied to fit the cell circuit model based on the remaining goals to find R_c_ and C_c_. From the extracted variables, the dielectric permittivity (*σ*) and conductivity (*ε*) of the cytoplasm were calculated using Equations (1) and (2), as seen in previous single-shell fittings [21].

(1) $$\varepsilon_{c}=\frac{2C_{c}}{3\pi r_{0}}$$

(2) $$\sigma_{c}=\frac{2}{\pi r_{0}R_{c}}$$

In this single-shell model, the average cell radius as determined by microscopic image analysis (*r*_0_) helps calculate the cytoplasmic dielectric permittivity (*ε_c_*) and conductivity (*σ_c_*).

### 2.6. Statistical Analysis

Statistical analysis was performed using the python (3.11.2) package scipy.stats (v1.10.1) to compare means using an unpaired Students’s *t*-test. Statistical comparison of distributions was accomplished using a Kolmogorov–Smirnov test. For both, a *p*-value of less than 0.05 was considered statistically significant.

## 3. Results

**Figure 3:**
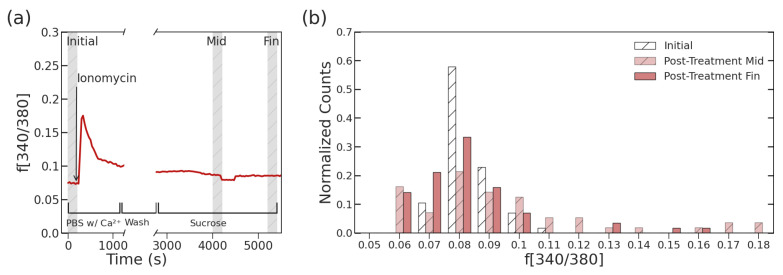
(**a**) Example f[340/380] curve over time with highlighted regions of interest and (**b**) normalized distribution of Ca^2+^-related fluorescence in pre-treatment (Initial) and post-ionomycin treatment cells at both the 30 min mark (Post-Treatment Mid) and 1 h mark (Post-Treatment Fin), as determined by f[340/380] (*n* = 89).

To determine the Ca^2+^ change, the fluorescence was monitored over time and the average f[340/380] in the periods prior to ionomycin addition and after the maximum determined experimental length of 1.5 h, based on viability in sucrose, as shown in Figure 3a. Although the cell slowly returns to the initial fluorescence level, there was still an elevation seen in many of the cells. After measuring these average values pre- and post-treatment for each individual treated cell, the distribution of was analyzed to determine how treatment changed the f[340/380] or relative Ca^2+^ concentration, which is shown in Figure 3b. The distribution of f[340/380] shows a slight peak shift higher after treatment with ionomycin and a notably wider distribution spread. The mean and standard distribution of f[340/380] signals from pre- and post-ionomycin treatment cells are 0.083 ± 0.008 and 0.084 ± 0.025, respectively. While the fluorescent signal mean is only slightly higher for the population after the treatment by ionomycin, there is a significant change in the shape of the distribution itself (*p* = 0.012). However, looking at the distribution in the middle of the time period during which electrical measurement occurs, there is a significant increase in mean to 0.102 ± 0.041 (*p* < 0.0009) and a significant shift in the distribution (*p* < 0.0008). This indicates that although the cells return eventually to the original Ca^2+^ level, there is an increase for the period wherein measurement occurs. By treating L6 skeletal muscle cells with ionomycin for a short period of time, Ca^2+^ mobility led to an extended increase in cytoplasmic Ca^2+^ by shifting the necessary electrical potential for gated channels [19]. Based on this previous work, membrane hyperpolarization is confined to the first three minutes of treatment wherein ionomycin can evoke transient outward currents such as Ca^2+^-activated K^+^ current (IK_Ca_), as demonstrated in our previous paper [19]. However, the previous work also found that stable membrane potential is re-established within 10 min. Due to this and the external addition of calcium, the slight shift in distribution results primarily from the internalization of externally available calcium. Despite the visible shift in distribution, there is still a significant overlap, something also seen in the distribution of later electrical measurements.

**Figure 4:**
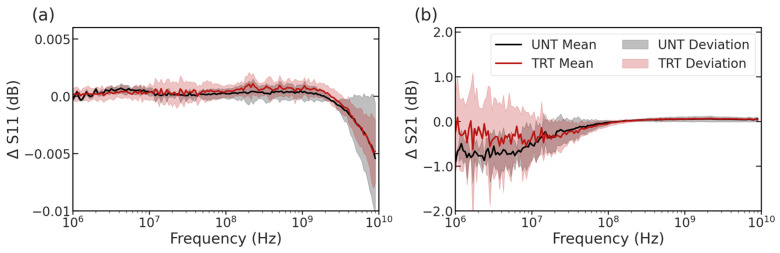
Average (**a**) ΔS11 and (**b**) ΔS21 spectra for untreated L6 cells (UNT) and cells treated with 1.5 µM Ionomycin (TRT) to increase cytoplasmic Ca^2+^ for 10 min. Shadows indicate deviation among measured cells while solid lines indicate the mean spectra (UNT, *n* = 52; TRT, *n* = 21).

Following the establishment of the ionomycin treatment as a method of increasing the intracellular Ca^2+^ in the L6 muscular cell line, broadband impedance spectroscopy was applied as a method for rapid cell capture and characterization of intracellular properties. The collection of spectra for each cell was examined, then compiled for statistical comparison of the mean curves and deviations in signals among treatment types, as shown in Figure 4. In this figure, the mean solid line is accompanied by a lighter shading region showing the deviation between electrically measured cells at each frequency. From this figure, while the overlap between groups is present at most frequency values, several distinct regions distinguish untreated L6 cells (UNT) and the ionomycin-treated cells (TRT) including the 10 MHz to 1 GHz range for the ΔS11 signal (Figure 4a) and the 1 MHz to 10 MHz range for the ΔS21 signal (Figure 4b). These ranges are relevant to the cytoplasm properties considering the scattering properties of most biological materials. At these frequencies, β-scattering is thought to dominate the electrical signal [16]. While visually distinct in several areas, most notably the MHz region, the differences are better characterized and explained by the use of circuit modeling to extract the compartment dielectric changes.

**Figure 5:**
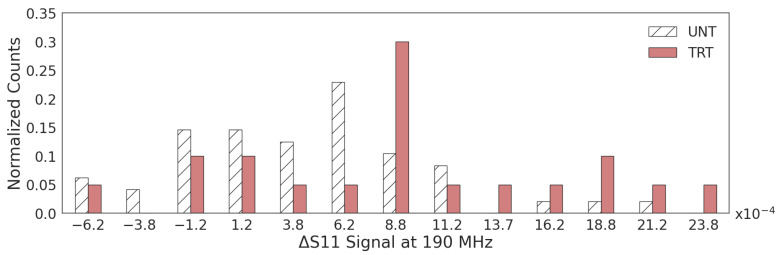
Normalized Δ S11 signal distribution for both untreated (UNT) and ionomycin-treated (TRT) cells when measured at 190 MHz.

The overlap due to the subtle differences between the UNT and TRT populations can also be visualized from the impedance measurements similarly to the fluorescent Ca^2+^ distribution, shown in Figure 3. Although the duration of electrical measurement is longer than that of calcium imaging, there was no observed signal drift when comparing cells measured at the beginning or end of the hour, indicating leakage current, if exists, is weak after cell wash. Thus, the influence of leakage current on cytoplastic impedance during the sensing period is neglected. Looking at one relevant frequency point at 190 MHz, the Δ S11 signal distribution looks similar to the spread seen in Figure 5, where the TRT population has a wider distribution, specifically in the increasing signal direction. The UNT population has a ΔS11 signal of 3.5 ± 3.3 × 10^−4^ compared to the TRT population, which had a significantly higher signal of 8.1 ± 4.5 × 10^−4^ when measured at 190 MHz (*p* = 0.021). At this frequency, there is a significant shift in distribution mirroring the Ca^2+^ shift, which indicates a sensitivity to ionic changes (*p* = 0.017). In the MHz range, the electrical signal can penetrate the cell membrane for measurements of the cytoplasm, and in the lower MHz range, there is minimal measurement interference from organelle structure or large molecules within the cell, making 190 MHz an ideal frequency to look at the dielectric properties of the cytoplasm itself [15,16].

**Figure 6:**
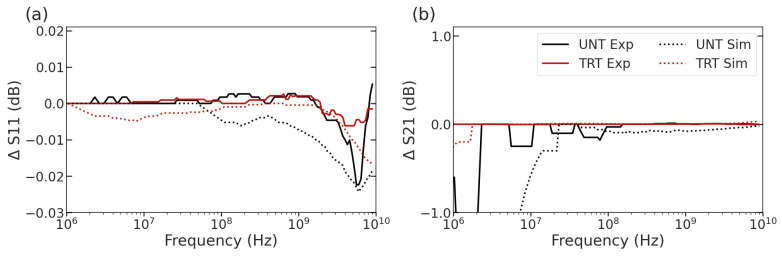
Experimentally collected spectra (solid) and simulated spectra (dashed) for both ionomycin-treated cells and untreated cells for (**a**) ΔS11 and (**b**) ΔS21.

To better understand the implications of the collected ΔS11 and ΔS21 spectra, a single-shell circuit model fits the electrical properties of the cytoplasm resistance (R_c_) and capacitance (C_c_). The circuit model relies on the assumption that the induced increase in Ca^2+^ has no effect on the membrane characteristics or the membrane returns to its typical homeostasis quickly. The assumption is supported by the gradual return of Ca^2+^ over time to the initial concentration as the homeostasis processes regulate the individual cells. By applying the model to each curve, the way properties impact the spectra can be examined. In the spectra found in Figure 6, representative cells from each treatment group are shown with simulations closely match the spectral shapes of the experimentally generated curves. The extracted values of these cells are roughly 0.3 MOhm for R_c_ for both treatment types and 3.89 fF and 3.76 fF for the C_c_ for the UNT and TRT cells, respectively. While the differences were minimal in the circuit values themselves, the dielectric parameters equate to have significantly different fitting values.

**Figure 7:**
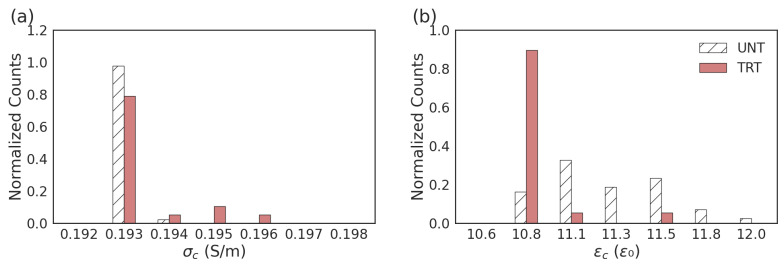
Normalized distribution of calculated single-shell model parameters for the (**a**) cytoplasmic conductivity (*σ_c_*) and (**b**) relative cytoplasmic permittivity (*ε_c_*).

Using the single-shell model and Equations (1) and (2), the fit resistance (R_c_) and capacitance (C_c_) of the cytoplasm can also be extended to calculations of the cytoplasmic permittivity (*ε_c_*) and conductivity (*σ_c_*). Based on the extracted parameters, the *σ_c_* for the UNT and TRT populations were 0.1929 ± 0.0002 S/m and 0.1933 ± 0.0008 S/m, respectively. Though small in magnitude, the TRT cells had a significantly higher conductivity (*p* = 8.3 × 10^−5^). The extracted ε_c_ of UNT cells was 11.3 ± 0.34 *ε*_0_ and had a significantly lower (*p* = 0.0063) average of 10.9 ± 0.13 *ε*_0_ for TRT. Looking at the distribution of extracted *σ_c_* and *ε_c_* in Figure 7, there are also significant differences in the distribution of fitted parameters between untreated and ionomycin-treated cells. Proportionally, a higher percentage of treated cells have a slightly larger conductivity and a smaller permittivity than the untreated cell distribution.

## 4. Discussion

In Figure 3b, the distribution of the fluorescence related to calcium peaks at a higher ratio and spreads wider after Ionomycin treatment, consistent with an increase of calcium due to treatment. Based on the known action of ionomycin as an electroneutral ionophore, the cells are expected to rapidly transport extracellular calcium inside, then recover normal cytosolic concentrations after the removal of the ionomycin [27,28]. Another common method of monitoring Ca^2+^ manipulation within cells is nanosecond pulsed electrical fields (nsPES), which deliver a small electrical stimulant to generate pores in the cell plasma membrane, depending on the signal magnitude and polarity [29]. Recent work in this field to look at Ca^2+^ increase within cells has shown that the presence of sucrose molecules can delay the swelling response typically associated with an increase in intracellular Ca^2+^; however, this can depend on the external concentration introduced and the voltage-gated channels present on the cell type of study [30,31]. As with our study, these show that while established in creating a gradient to increase cytosolic Ca^2+^, there are less understood effects on deeper membranes and compartments within the cell after treatment [32]. While the calcium change is the main factor associated with ionomycin treatment, several long term changes can be noted, including expression of IL-6 [33,34] or CAIII [35]; however, these are unlikely to cause an impact in the short term. Due to the rhythmic nature of Ca^2+^ function in cells, regulation of levels in the cytosol is carefully controlled by several proteins through storage and release in the sarcoplasmic reticulum. Based on this crucial system, it is anticipated that while the overall distribution increased, some cells returned to fluorescence consistent with their initial values. The rapid peak of fluorescence and accompanying plateau is consistent with previous work observing the rapid flux of cytosolic calcium before slower recovery after treatment with ionomycin [36].

Similarly, looking at the electrical measurements, the increase is consistent with cytosolic Ca^2+^ increase and the extracted values are consistent with previous measurements of similar cellular changes. The values from the UNT cells are comparable in scale to the previously published fitting value of 0.22 S/m and 9.49 *ε*_0_ [21]. The statistical comparison between these values for the UNT (*n* = 51) and TRT (*n* = 20) cells shows that a significant increase exists for conductivity and a significant decrease exists for permittivity. The small magnitude of change is consistent with the expected pattern of recovery after the removal of ionomycin transport complexes. For the maintenance of cell viability, the change in calcium while established would be minimal in a healthy population. The observed change in conductivity and permittivity is consistent with an increase in ions, making a more even distribution of charge throughout the cell cytoplasm. It has been noted earlier that based on the initial Ca^2+^ imaging data, not every ionomycin-treated cell sustains the increased intracellular Ca^2+^ concentration, leading to the significant overlap between the UNT and TRT groups. It is also important to note that while this work focuses on the cytosolic changes, the homeostasis of calcium management also occurs in the sarcoplasmic reticulum. However, due to the broadband nature of the reported measurements, changes in other compartments are captured in a wide range of frequencies.

The previous work posits oxidative damage to the mitochondria and flooding of intracellular reactive oxygen species (ROS) and Ca^2+^ prior to the start of apoptosis when exposed to chronic oxidative stress [37]. Physiologically, there is a well-established link between Ca^2+^ levels in skeletal muscle and the ability to maintain the balance of ROS and mitigate the effects of oxidative stress. Not surprisingly, the spectra alteration observed here from intracellular Ca^2+^ elevation, an increase in ΔS11 in the MHz range followed by a dip in the GHz range and a less negative value of ΔS21 in the kHz range, is comparable to those from L6 cells exposed to long-term oxidative stress [21]. The previous work identified Ca^2+^ as a key factor differentiating cells that experienced exposure to oxidative stress, while in this work, the ability to differentiate calcium through modelling shows the milder but noticeable contributions Ca^2+^ makes to the internal dielectric properties. Alternatively, work using the ratio of impedance at 1 MHz to 300 kHz to characterize individual cell opacity also showed the ability to measure the changes in neutrophils due to calcium ionophore exposure [38]. This work has a higher throughput and therefore, sample size; however, it is limited to blood cells and limited to analysis of size and opacity to characterize the populations studied. By measuring a full spectrum of frequency values rather than relying on a smaller number of frequencies, this work shows the capture of dielectric properties representing complex and multifaceted changes due to elevated Ca^2+^ levels induced by ionomycin.

The ability to identify cytoplasmic Ca^2+^ changes has the potential to aid in improving our understanding of many associated skeletal muscle diseases such as DMD, cachexia, and the process of sarcopenia development [2]. Considering that earlier published work also showed that calcium influx is an important factor in the way long-term oxidative stress changes the electrical properties of muscle cells, the results from this study are not meant to differentiate between calcium mismanagement and oxidative stress, but rather explain the contribution calcium mismanagement might have towards the oxidative stress responses previously observed. This work is also limited by the selectivity of the electrical spectroscopic method to define specific molecular or ionic contributors with certainty. Due to the complexity of ion management inside cell models, while the treatment is intended to alter intracellular Ca^2+^ concentration, the effects of other ions or molecules may also contribute to the observed changes in the electrical signal. This limitation of ion sensing selectivity has been reported in an aqueous solution at high frequencies [39,40]. Because electrical measurements are widely non-specific and oxidative diseases have complex and multifaceted effects on muscle cells, the objective is to broaden understanding of how these effects manifest electrically. As ionomycin treatment is known to alter ion concentration inside cells without inducing oxidative stress, this study allows us to focus the manifestation of calcium imbalance in cellular impedance. Additionally, while this work focuses on skeletal muscle, the potential to monitor neuron resting and altered Ca^2+^ concentration have implications for many more diseases [41,42]. Typically, measurement of Ca^2+^ in vivo relies on the inclusion of fluorescent agents such as the Fura-2 in this work to look at cytosolic Ca^2+^ or Mag-Fluo-4 to look at Ca^2+^ in the endoplasmic reticulum [20,43]. However, these options require cell labeling and complex treatment processes, both of which are avoided by electrical measurement. The electrical system presented can offer a wider view of the individual cell dielectric properties at multiple frequencies, more rapid measurement, and fewer resources required. There is a need to demonstrate a realistic sensitivity to biological levels of cytoplasm Ca^2+^ for disease diagnosis or monitoring applications, which shapes our approach in the future. Going forward, the spectral changes seen in this work will be used in a further study of ME/CFS clinical samples looking at how skeletal muscle electrical properties at a variety of frequencies can be correlated with biological changes to further our understanding of this rare disease.

## 5. Conclusions

Based on the electrical measurements and corresponding extracted parameters, there is a subtle change in MHz and GHz spectral patterns that can be correlated with fluorescent-image-based cytoplasmic Ca^2+^ levels. The electrically measured differences can be further described by changes to the dielectric parameters of cytoplasmic permittivity (*ε_c_*) and conductivity (*σ_c_*). In this work, it was found that increased cytoplasmic Ca^2+^ concentration can be associated with a significant increase in cytoplasmic conductivity and a decrease in cytoplasmic permittivity. This monitoring system improves the depth of information available about intracellular conditions and ion study in the cytoplasm. The work presented here is limited by the lack of comparison with true concentration correlation and selective sensing of the measurement system to particular ions; therefore, further exploration is necessary to develop a true system for disease monitoring. That being said, understanding these Ca^2+^ levels can help generate understanding and evaluate skeletal muscle disease progression and treatment effectiveness. In addition, by modeling these changes in the context of pre-evaluated oxidative changes, important deductions can be made about how different properties associated with ME/CFS contribute to an overall electrical profile to move toward a unique and rapid diagnostic tool.

## Figures and Tables

**Table 1:** Goal and weight parameters (W) assigned to automated simulation circuit models in Advanced Design System (ADS) software and variables tuned in each optimization.

Optimization	Circuit Design	Sweep Goal 1			Sweep Goal 2			Variable
		*Target*	*Freq*	*W*	*Target*	*Freq*	*W*	
Optim 1	Background	ΔS11	9 kHz–9 GHz	1	ΔS21	9 kHz–9 GHz	1	C_g_
								C_s_
		ΔS11	0.9 MHz–9 GHz	2	ΔS21	9 kHz–9 MHz	2	R_0_
								Z_0_
								Z_g_
Optim 2	Single-Shell	ΔS11	9 kHz–9 GHz	5	ΔS21	9 kHz–9 GHz	1	R_c_
		ΔS11	0.9 MHz–9 GHz		ΔS21	9 kHz–9 MHz		C_c_

## Data Availability

Data available upon request.
